# Predictors of Dental caries among children 7–14 years old in Northwest Ethiopia: a community based cross-sectional study

**DOI:** 10.1186/1472-6831-13-7

**Published:** 2013-01-18

**Authors:** Fenta A Ayele, Belaynew W Taye, Tadesse A Ayele, Kassahun A Gelaye

**Affiliations:** 1 Public health specialist, Amhara National Regional State, Amhara, Ethiopia; 2 Department of Epidemiology and Biostatistics, Institutes of Public Health, University of Gondar, P. O. Box 196, Gondar, Ethiopia; 3 Department of Environmental and Occupational Health and Safety, Institutes of Public Health, University of Gondar, P. O. Box 196, Gondar, Ethiopia

**Keywords:** Dental caries, Mouth rinsing, Predictors, Northwest Ethiopia

## Abstract

**Background:**

Dental caries in children remains a significant public health problem. It is a disease with multifactorial causes. The aim of the study was to assess the prevalence and associated factors of dental caries among children between 7 to 14 years old.

**Methods:**

A community based cross-sectional study was conducted in Gondar town from June 2011 to September 2011. A total of 842 children were involved in the study. Multi-stage sampling technique was used to select the children. Pretested and structured questionnaires were used to collect data from mothers. Clinical examination of children was done using dental caries criteria set by world health organization. Data were entered, cleaned and edited using EPI Info version 3.5.1 and exported to SPSS version 16.0 for analysis. Binary multiple logistic regression analyses was applied to test the association.

**Results:**

Four hundred sixty three (55%) children were females. The prevalence of dental caries was 306(36.3%).The educational status of children’s father (AOR=0.3, 95%CI, 0.17, 0.80), monthly household income (AOR=0.59, 95%CI, 0.01, 0.45), regular teeth brushing (AOR=0.08, 95% CI, 0.03, 0.20) and using mouth rinsing (AOR=0.40, 95% CI, 0.2, 0.80) were found statistically significantly associated with dental caries.

**Conclusions:**

Dental caries were high among children in Gondar town. Low socioeconomic status and poor oral hygiene practices were the influencing factors for dental caries. Oral hygiene, dietary habits and access to dental care services are supreme important for the prevention of the problem.

## Background

Dental caries are one of the public health problems in both developed and developing countries [1]. In developing countries, dental caries is increasing gradually due to the growing consumption of sugary substances, alcohol, substance abuse, cigarette smoking, poor oral care practices and inadequate health service utilization [2]. Dental caries result from dental plaque (bacteria) present in and around the teeth for long time. The frequent intake of sweets, dry mouth, and poor oral hygiene increase the chances for cavities [3]. The timing of the tooth eruption, the time span of the harmful dietary habit and the type of muscle movements during sucking and swallowing are the common factors of dental caries. The early childhood caries pattern changes at age three and begins to affect the first and second primary molars [4,5].

In Israel, 57% immigrants had a "bad" health status of their teeth, 56% had gum problems, and 60% suffered from tooth ache [6]. The dental caries get increased with increasing age [7,8]. A study done in Northwest Ethiopia showed that the overall prevalence of dental caries was 47.1% [9]. In Ethiopia, prevention and treatment of oral diseases receive little attention. Despite the fact that oral diseases are affecting majority of the Ethiopian children, much is not known about the extents and factors influencing the occurrence of dental caries and oral care practices and health care seeking behavior in most parts of the country particularly in the study area. This paper aimed to address this gap by attempting to identify predictors of dental caries and barriers to oral care practices. Therefore, the results of this paper may draw the attention to public health decision makers, ministry of health, nongovernmental organizations and other stake holders to revise their policies and programs.

## Methods

A community based cross-sectional study was conducted from June 2011 to September 2011 to assess the prevalence and associated factors of dental caries among children at Gondar town. Gondar town is 737 kilometers away from the capital, Addis Ababa. According to 2011 Central Statistical Agency of Ethiopia (CSA) estimate, the town had a total population of 244,583 of whom children under 15 years of age were 104,192 [10]. The town has 12 urban and 12 rural *kebele* administrations. It has two government hospitals, seven health centers and 37 private clinics and one private hospital [11].

Children between 7 to 14 years of age and living in Gondar town were included in the study. Sample size was calculated using single population proportion formula with an assumption of 95% confidence level, 5% degree of precision, proportion of dental careis, 47.1% and design effect of two. Ten percent non- response rate was added to make the final sample size 842. Multistage random sampling was employed to select the study subjects. Among twelve urban *kebele* administrations six *kebele* were selected randomly and then 62 ‘*ketenas’,* the lowest administration, were selected using simple random sampling technique from each *kebele*. The sample size was allocated proportionally according to the size of children in each ‘*ketena’.* Number of children and their name lists were taken from health posts and then children were selected randomly.

Pretested and structured questionnaires that included socio-demographic characteristics, dietary habit, health care seeking behavior towards oral health problems and oral care practice were used for data collection. The aim of the pretest was to increase the validity and reliability of data collection tools. Hence, data collection tools were pretested in other area which had a similar setup of the study area ahead of the actual data collection. After the pretest, we did amendments on the final version questionnaire based on the feedback we obtained from the pretest. Dental examination was carried out to all randomly selected children by dental doctors to identify the presence of dental caries cases. The dental examination was done in day light. Disposable wooden spatulas were utilized to check the presence of decay.

Trained six data collectors for data collection and three dental doctors for physical examination were involved. Prior to the study, data collectors were given two days intensive training on dental caries assessment based on world health organization (WHO). The dental caries diagnosis protocol was obtained from WHO dental caries diagnosis guideline. The dentists or dental doctors were trained and practiced on pretest to follow and keep up uniform and standard dental caries assessment on children.

Caries was recorded as being present when a lesion in a pit or fissure or on smooth tooth surface had a detectable softened floor, undermined enamel or softened wall. A filled tooth also included in this category when it contains one or more restorations and one or more areas that are decayed. When any doubt existed, caries was not recorded as present. Tooth was considered missing because of caries if a person gave a history of pain and/or presence of cavity prior to extraction. Incomplete questionnaires were refilled during field work. Data were entered, cleaned and edited in EPI Info version 3.5.1 and exported to SPSS for windows version 16.0 for further analysis. Bivariate and multivariate analyses were employed. Odds ratio with 95%CI was computed to assess the presence and degree of association between dependent and independent variables. A p-value ≤ 0.05 was considered statistically significant in this study.

Ethical clearance was obtained from University of Gondar College of medicine and health sciences institutional review board. Official communication was made between Gondar city mayors. A written consent was obtained from parents before interview and examination. The data were collected anonymously. Cases of dental caries were referred to University of Gondar referral hospital dental clinic for treatment.

## Results

A total of 842 study subjects were included in the study. Four hundred sixty three, (55%) were females. Five hundred seven (60.2%) of the children were in the age group of 7 to 10 years. The mean (±SD) age of the children was 10.05(± 2.12) years. The majority, 727(86.3%) of children, were Amhara in ethnicity and 692(82.2% were Orthodox Christian in religion.

Two hundred seventy three (32.4%) mothers attained grades 7 to 12 and 268 (31.8%) of fathers were above grade 12 in their educational status. The majority, 593(70.4%) of children were in first cycle education. Three hundred sixty three (43.1%) families earned below 27 US Dollar per month (Table 1). 

**Table 1:** Socio-demographic characteristics of children and their parents at Gondar town, Northwest Ethiopia, September 2011

**Variables**	**Frequency**	**Percentage**
**Age**		
7-10	507	60.2
11-14	335	39.8
**Sex**		
Male	379	45
Female	463	55
**Ethnicity**		
Amhara	727	86.3
Tigrie	73	8.7
Other	42	5
**Religion**		
Orthodox	692	82.2
Muslim	119	14.1
Protestant	13	1.5
Catholic	11	1.3
Other	7	0.8
**Marital status of parents**		
Married	559	66.4
Single	25	3.0
Divorced	142	16.9
Widowed	116	13.8
**Educational status of mother**		
Illiterate	234	27.8
Can read and write	51	6.1
1-6 grade	155	18.4
7-12 grade	273	32.4
>12 grade	129	15.3
**Educational status of father**		
Illiterate	112	13.3
Can read and write	94	11.2
1-6 grade	125	14.8
7-12 grade	243	28.9
>12 grade	268	31.8
**Educational status of child**		
0-4	593	70.4
5-8	249	29.6
**Monthly Income (US dollar)**		
<27	363	43.1
28–56	239	28.4
57–84	66	7.8
85–112	93	11.0
113–167	46	5.5
>168	35	4.2

### Food consumption pattern and dietary habits of children

More than half, 462(54.9%), of the children had three times meal schedule per day. Six hundred thirty six, (75.5%) had breakfast bread with tea, 799(94.9) had lunch *injera* with sauces and 524 (62.2%) ate snacks three times per day. Seven hundred thirty nine (87.8%) of the subjects drunk tea with sugar, 478 (56.8%) of the children used to drink soft drinks and 722 (85.7%) used to consume sweet foods and drinks. Among the sweet food consumers, 199 (23.6%) took daily (Table 2). 

**Table 2:** Food consumption pattern and dietary habits of children aged between 7–14 years, Gondar town, North West Ethiopia, September 2011

**Variables**	**Frequency**	**Percent**
**Frequency of meal**		
Once per day	5	0.6
Twice per day	43	5.1
Three times per day	462	54.9
Four times and above	332	39.4
**Type of food for breakfast**		
Bread with tea	636	75.5
Bread with milk	45	5.3
*Injera* with *wot*	140	16.6
*Kinche*	21	2.5
**Type of food for lunch**		
Bread with tea and milk	22	2.6
*Injera* with *wot*	799	94.8
*Kinche* and *pourage*	21	2.6
**Snack frequency**		
Three times per day	524	62.2
Twice per day	34	4.0
Once per day	197	23.4
Occasional	87	10.3
**Consumption of sugared tea**		
Yes	739	87.8
No	103	12.2
**Consumption of sugared coffee**		
Yes	95	11.3
No	747	88.7
**Consumption of soft drinks**		
Yes	478	56.8
No	364	43.2
**Consumption of sweet foods and drinks**		
Yes	722	85.7
No	120	14.3
**Frequency of sweet foods and drinks**		
Daily	199	23.6
Once per week	74	8.8
Occasionally	449	53.3

### Health seeking behaviors of parents and practices of dental care

About 284(33.7%) of parents/care takers accepted that their children had problem of dental caries. Only one hundred ninety one (67.3%) of parents/care takers took their children to health institutions for medical help.

**Figure 1:**
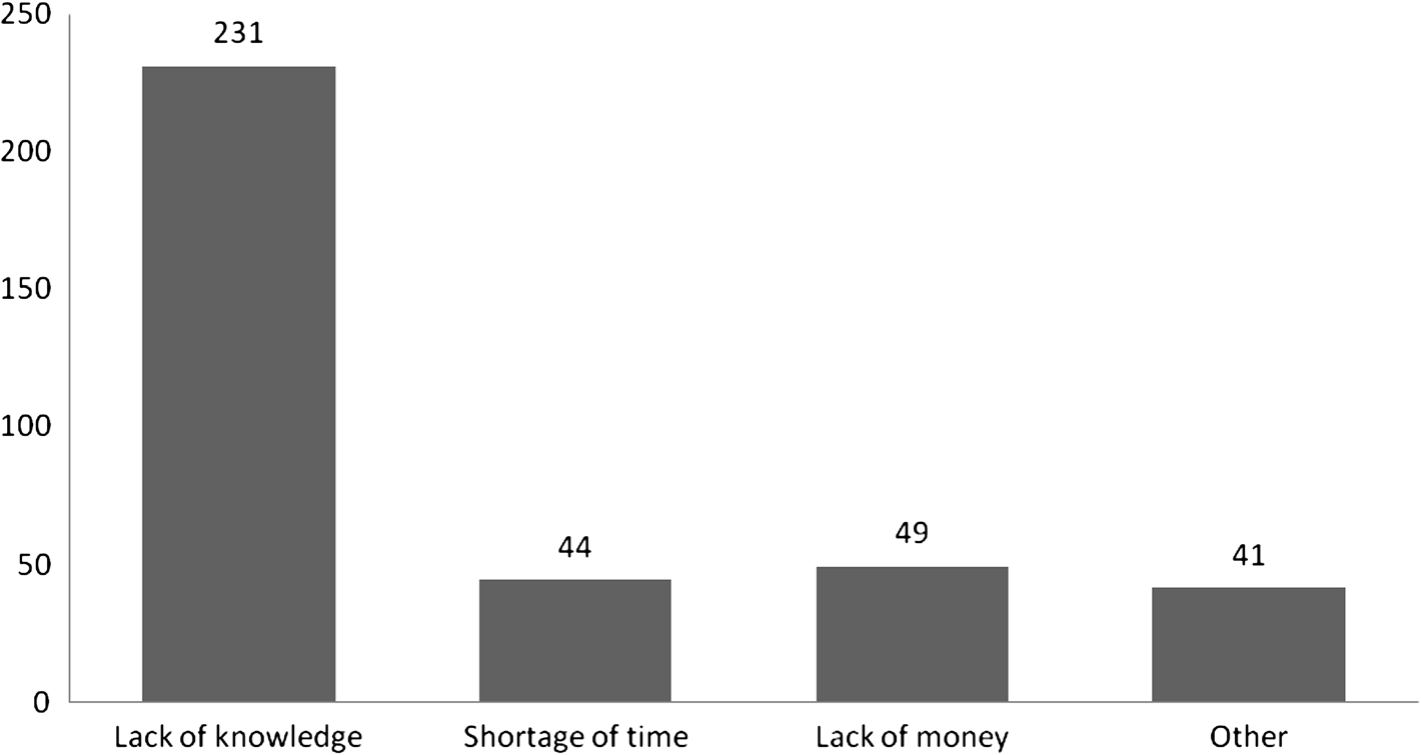
Mothers reasons for not brushing their children’s teeth at Gondar town, Northwest Ethiopia, September 2011.

Four hundred seventy seven (56.7%) children brushed their teeth, among these 189(38.8%) of the children brushed their teeth daily. Lack of knowledge 231(63.3%), time 44(12.15) and money 49(13.4%) were the common reasons for not their children brushed their teeth (Figure 1). 

### Dental caries

All types of decayed teeth were identified in 306(36.3%) children and the majority, 53(6.3%) had incisors total decayed. All types of missed teeth were present in 135 (16.0%) children and molar total missed were the largest 44(33.33%) found in children. No filled cases were reported at all (Table 3). 

**Table 3:** Dental cases of Gondar town children aged 7-14 years, North West Ethiopia, September 2011

**Characteristics**	**Frequency**	**Percent**
**Decayed teeth (all types)**		
Yes	306	36.3
No	536	63.7
Incisors total decayed	53	17.3
Canines total decayed	44	14.4
premolars total decayed	105	34.3
Molar total decayed	172	56.2
**Missed teeth (all types)**		
Yes	135	16.0
No	707	86.0
Incisors missed	18	13.3
Canines missed	32	23.7
Premolars missed	41	30.4
Molars missed	44	33.3
**Place of Treatment**		
Health Institute	192	67
Pharmacy	16	6
Traditional	12	4
Don’t go anywhere	65	23

### Factors associated with dental caries problem among children

There was statistically significant association between dental caries and educational status of the father (AOR = 0.37, 95%CI, 0.17, 0.80). Dental caries among children whose father were above grade 12 were 63% times at a lower risk compared to illiterates. Dental caries were lower among families of children who earned greater than 168 US Dollar per month as compared to those earn less than 27 US Dollar per month (AOR = 0.006, 95%CI, 0.074, 0.47). Children who had cleaned their teeth were 99.4% times less likely to have caries as compared to those who did not clean (AOR = 0.078, 95%CI, 0.03-0.20). The odds of having dental caries was also lower in children who have been rinsing their mouth after meal as compared to those who do not rinse (AOR = 0.399,95%CI,0.198,0.804) (Table 4). 

**Table 4:** Factors associated with dental caries among children aged 7-14 years at Gonda town, Northwest Ethiopia, September 2011

**Characteristics**	**Decayed teeth**		**COR ( 95% CI)**	**AOR ( 95% CI)**
	**Yes**	**No**		
**Ethnicity**				
Amhara	272	455	1.00	1.00
Tigray	23	50	0.769 (0.459 ,1.289)	0.705 (0.328, 1.517)
Kimant	7	29	0.404 **(0.175 ,0.934)**	0.627 (0.075, 1.947)
Other	4	2	3.346 (0.609 ,18.38)	1.092 (0.142, 8.401)
**Educational status of father**				
Illiterate	44	68	1.00	1.00
Read & write	38	56	1.049 (0.599,1.836)	1.185 (0.508,2.766)
1-6grade	54	71	1.175 (0.700,1.974)	1.378 (0.615,3.088)
7-12grade	95	148	0.992 (0.627,1.569)	0.894 (0.436,1.833)
>12grade	75	193	0.601 **(0.378,0.955)**	0.369 **(0.170, 0.803)**
**Economic status**				
< 28	157	206	1.00	1.00
29 – 56	93	146	0.836 (0.599,1.166)	0.875 (0.51, 1.486)
57 – 84	20	46	0.570 (0.324,1.003)	1.291 (0.564, 2.955)
85 – 112	24	69	0.456 **(0.274,0.759)**	0.628 (0.307, 1.286)
113 – 167	8	38	0.276 **(0.125,0.609)**	0.521 (0.175, 1.527)
>168	4	31	0.169 **(0.059,0.490)**	0.059 **(0.008, 0.447)**
**Snack frequency**				
Three/day	178	346	0.729 (0.459,1.158)	1.366 (0.613, 3.045)
Twice/day	11	23	0.678 (0.294,1.563	1.251 (0.326, 4.791)
Once/day	81	116	0.989 (0.0.593,1.651)	1.018 (0.429, 2.417)
Occasional	36	51	1.00	1.00
**Sweet foods and drinks**				
Yes	282	460	1.941 (1.199,3.144)	1.814 (0.908,3.626)
No	24	76	1.00	1.00
**Sugared coffee**				
Yes	175	303	1.00	1.00
No	131	233	1.027 (0.774,1.364)	0.859 (0.449, 1.643)
**Cleaning teeth**				
Yes	120	357	1.00	
No	186	179	0.73 **(0.54, 0.97)**	0.077 **(0.030-0.201)**
**Rinsing mouth**				
Yes	260	49	1.00	1.00
No	46	40	0.46 **(0.291,0.715)**	0.399 **(0.198-0.804)**
**Soft drinks**				
Yes	224	60	1.00	1.00
No	82	476	21.67 (14.98, 31.34)	1.026 (0.714, 1.871)

## Discussion

The trends of dental caries in developing countries are gradually increasing. This study attempted to assess the prevalence and associated factors of dental caries among 7–14 years old children in Gondar town. The prevalence of dental caries found in this study was higher than a cohort of Ethiopian immigrants to Israel [6]. However, it was lower than a study done in *kimer Dengay*, Ethiopia [9]. The difference in the later could be due to difference in access to awareness on oral hygiene as this study was undertaken in urban setting.

In this study, educational status of children’s father was found to be statistical significant in different levels of dental carries. Hence, as education level of the father increases the probability of dental caries get reduced. This finding was also supported by similar findings [12-14]. There was also significant difference between monthly income of the households and dental caries. As income of the families increasing, children are less likely to be affected by dental decays. This could be elaborated that those who have better monthly income can have potential to buy tooth cleaning materials and well awarded for changes and have better health care seeking behavior. Similarly studies revealed that low monthly income were found to be more affected by teeth decays [14,15].

This study revealed a strong positive association between caries development and tooth brushing practices. The more frequent brushing is performed the less caries children experiences [13]. Study done in Brazil also stated that children who started oral hygiene earlier presented with a lower prevalence of early childhood dental caries [15].

Mouth rinsing was significantly associated with caries. Individuals that rinse their mouth with water after meal were less likely to be attacked by dental caries than those who did not rinse. This could be due to the washing away of sugary food substances from the teeth; therefore, micro-organisms cannot get enough time for multiplication and growth and no acid production that causes caries development [16].

Molars decayed and premolars were the most frequently affected teeth by dental caries. The reason could be majority of the food was grinded with these teeth and both of them have very large surface area in which the food particles accumulated and harbor micro-organisms to proliferate. As a result, these acid producing bacteria might affect the enamel of the tooth which later went into decayed teeth. On the other hand the canines and incisors had the lowest decayed percentage among the four types of teeth. This could be explained by the fact that both of them are protected from direct exposure to acidic food by the tongue and less chance chewing food materials, also they are close to the sublingual salivary gland duct where it helps in diluting the acidic environment around the lower incisors.

Dental caries cases were identified only with clinical diagnosis, difficulty of radiological examination at field level might reduce the actual magnitude of the problem. Sweet food items and drinks were assessed by the usual patterns and/frequency of intake but the amount and the duration of intake was not well assessed.

## Conclusion

Prevalence of dental caries was relatively higher and found public health problem. Lower socioeconomic status, lower father's educational level and poor oral hygiene practices were associated factors for children dental caries. Oral hygiene, dietary habits and access to dental care services are supreme important for the prevention of the problem.

## Competing interests

The authors declare that they have no any conflict of interests.

## Authors’ contribution

FA wrote the proposal, participated in data collection, analyzed the data and drafted the paper. BW, KA and TA approved the proposal with some revisions, participated in data collection and analysis. All authors participated in the preparation of the manuscript and approved the final manuscript.

## Pre-publication history

The pre-publication history for this paper can be accessed here:

http://www.biomedcentral.com/1472-6831/13/7/prepub
